# Spotted Fever without Cerebro-Spinal Meningitis

**Published:** 1865-08

**Authors:** James J. Levick

**Affiliations:** Physician of Pennsylvania Hospital


### Miscellaneous.

### Spotted Fever without Cerebro-Spinal Meningitis.

*By James J. Levick, M. D., one of the Physicians of Pennsylvania Hospital.*

At the present time, when the true nature of the so-called "spotted fever" is exciting much discussion, both at home and abroad, the following notes of a case of this disease coming under the writer's care may not be uninteresting:

During the prevalence of the epidemic of spotted fever in this city in the early part of last year I was called by my friend, Dr. Scholfield, to see Ellen C———, a tall and robust Irish woman, residing near Eighth and Filbert streets. She had been detained at a restaurant, where she was employed as cook, until a late hour of the preceding night. She went to bed at 2 A. M., apparently as well as usual. During the night she was seized with a chill, nausea and vomiting. This latter continued throughout the night. She was seen by Dr. Scholfield at about 10 o'clock in the morning, and a few hours later by the writer, and presented the following appearance: she was sitting up, gave the history of her attack as noted above, said she had no pain in her head, but complained of severe pain in the region of the heart and epigastrium. The conjunctivæ were injected, and the pupils promptly responded to the stimulus of light. No pulse could be felt at the wrists or at the bend of the arm; the heart was acting feebly and irregularly.

The face presented an extraordinary appearance. On each cheek there were large, dark, purple extravasations of blood, varying from half an inch to an inch in size, while the interspaces were dotted with small petechiæ. On closer examination almost every part of the surface of the body was found to be thus spotted, the spots varying from patches of two inches in width to points not larger than the head of a pin.

Although regarded by us as too ill to be disturbed her removal was insisted on by the people of the house, and the woman was soon after sent in a carriage to the Pennsylvania Hospital. When admitted she was unconscious and moribund, with great lividity of the surface, and she died at 2 P. M., just twelve hours after she had gone to bed apparently in her usual health.

From the time she was first seen by Dr. Scholfield she had taken freely of brandy and quinia.

The *autopsy* was made next day at 11 A. M., in the presence of Drs. Gerhard, Hartshorne, Morton, the resident physician, and the medical class of the hospital. The appearances presented were as follows: *Exterior.* Rigidity well marked. The entire surface of the body, excepting those parts on which it rested, was of a dull livid color. There were patches, as before described, on the face, chest, and abdomen, and numerous petechiæ on the legs. On cutting through the scalp there was an escape of dark fluid blood with which the vessels were turgid. A large echymosis was found on the left temporal bone, and smaller ones on other parts of the cranium. The meningeal vessels were filled with black blood. The most careful examination failed to detect any evidence of inflammation either in the substance of the *brain* or in its membranes. The *spinal cord* was removed in its entire length, and was examined both by the unassisted eye and with the microscope. It was of a firm consistence, and in every way free from disease.

The *lungs* contained a large quantity of dark fluid blood. There were blood stains on the pleuræ and on the arch of the aorta.

The *heart* had undergone a slight, fatty degeneration. It contained a large quantity of fluid blood; was free from coagula, excepting three soft clots about the size each of a pea. The *spleen* was firm and of the usual size. The *liver* congested and fatty. The *stomach* and *intestines* both on their outer and inner coats were dotted with innumerable blood spots. A large extravasation was found on the *pancreas*, and one entirely covering the summit of the *uterus*. In the *ovaries* several vesicles were found filled with black blood. A few spots were seen on the bladder, and a large number on and in the kidneys.—[*Medical and Surgical Reporter*.

JUNE 25, 1865.

